# Efficacy and Tolerability of Fitostimoline in Two Different Forms (Soaked Gauzes and Cream) and Citrizan Gel in the Topical Treatment of Second-Degree Superficial Cutaneous Burns

**DOI:** 10.1155/2011/978291

**Published:** 2011-04-04

**Authors:** Patrizia Martini, Carlo Mazzatenta, Giorgio Saponati

**Affiliations:** ^1^‘‘U.O. di Dermatologia”, “Campo di Marte” Hospital, Azienda USL 2, 55100 Lucca, Italy; ^2^Ispharm s.r.l., Via Dorati 117, 55100 Lucca, Italy

## Abstract

A total of 227 patients (mean age 41.3 years, 52% females) with at least one second-degree superficial cutaneous burn of thermal origin of a smallest transverse diameter ≥20 mm and a largest transverse diameter ≤90 mm were randomised to receive the topical application of aqueous extract of *Triticum vulgare* (Fitostimoline) in two different forms (soaked gauzes and cream) or catalase of horse origin in form of gel (Citrizan Gel), given up to healing or to a maximum of 20 days. The rate of lesion healing at end of study was significantly higher in patients treated with Fitostimoline (gauzes 97.3%, cream 91.5%) than in those receiving catalase (84.5%). The pooled Fitostimoline groups were also significantly more effective than catalase gel in reducing total symptoms score, pain at medication, pain at rest, and burning at end of study. Both formulations of Fitostimoline and catalase gel were well tolerated in terms of adverse effects in the site of application.

## 1. Introduction

The primary objectives of the treatment of second-degree superficial cutaneous burns are the maintenance of a microenvironment favourable for the cellular regeneration, the removal of the necrotic tissues and the prevention of bacterial proliferation, which interfere with the healing processes [1]. The local treatment of lesions is targeted at maintaining a wet microenvironment and at stimulating the formation of a well-vascularised granulation tissue, and the reepithelialisation of the lesion while counteracting the development of microorganisms, which is able to delay or prevent the biological phenomena of cicatrisation and reepithelialisation [2].

A variety of topical measures that ensure the protection of the burn and hence the anatomic-functional recovery of the affected skin are actually available; however, not all of them have in their composition factors that are able to stimulate the reparation [3]. Irrespectively of the preparation used in the local management of burn, the opinion of not proceeding with the replacement of the medication for more than once daily, to interfere as less as possible with the tissue reparation dynamic, is widely agreed [4]. In standard conditions and in absence of complications, the period of reepithelialisation of a medium-size second-degree superficial burn is approximately of two weeks [5].

The Fitostimoline in form of soaked gauzes and cream are widely used from some decades in the treatment of cutaneous lesions in which a stimulation of repairing processes (e.g., ulcerative-dystrophic damages, burns, delay in cicatrisation) is needed, and their place in therapy is well recognised [6, 7]. The aqueous extract of *Triticum vulgare*, the active ingredient of Fitostimoline-based products, determines a marked acceleration of tissutal repairing processes, stimulates chemotaxis and the fibroblastic maturation, and significantly increases the fibroblastic index, which are crucial points in the repairing processes [8]. These activities find an experimental confirmation both in the accelerated protein synthesis and in the enhanced ability of captation and incorporation of marked proline from tissues. Furthermore, the presence of 2-phenoxy ethanol in the product ensures an efficient antiseptical action [9, 10].

In this comparative study, two different formulations of Fitostimoline for topical use, that is, soaked gauzes and cream, were tested in the treatment of second-degree superficial cutaneous burns of thermal origin. A topical specialty indicated in the topical treatment of burns, sores and ulcers, which contains catalase of horse origin, has been chosen as reference drug.

## 2. Methods

### 2.1. Patient Population

The study protocol included patients of both sexes aged 18 to 70 years, presenting a second-degree superficial cutaneous burn of thermal origin started no more than 36 hours earlier. To be eligible for the study, the lesion was required to have a smallest transverse diameter ≥20 mm and a largest transverse diameter ≤90 mm (in the case of multiple lesions, the largest lesion that satisfied the inclusion criteria was taken into account). Patients with any of the following conditions had to be excluded from study participation: concomitant presence of third-degree burns; multiple lesions with total involvement >10% of body surface; burns localised at the level of head, neck, face, palmar or plantar surfaces, genital areas, peri-orifice zones, interdigital zones; presence of other important medical conditions (e.g., uncontrolled diabetes mellitus, severe hepatic or renal insufficiency, obstructive chronic arteriopathies involving the affected area of the study lesion, uncontrolled arterial hypertension, neoplastic diseases, or other concomitant serious infections); treatment with antineoplastics, immunosuppressants, or corticosteroids; pregnant or breastfeeding females, or females at risk of pregnancy.

### 2.2. Study Design and Treatments

The study plan included a baseline visit, in which patients eligible for the study were randomly assigned to receive one of the following three treatments: Fitostimoline soaked gauzes (Fitostimoline garze impregnate, Farmaceutici Damor S.p.A., Italy), Fitostimoline cream (Fitostimoline crema, Farmaceutici Damor S.p.A., Italy), and equine catalase (Citrizan gel IDI Farmaceutici S.p.A., Italy). Fitostimoline soaked gauzes were applied onto the lesion previously deterged with sterile saline solution and/or disinfected with H_2_O_2_; the medicated gauze was then covered with a sterile gauze (and, possibly, with cotton wool in the case of particularly secreting lesions) and bandaged. If required by the lesion size, the medicated gauze could be wound into itself. In the case that the roof of the bulla was intact, it had not to be opened. In patients treated with Fitostimoline cream or with catalase gel, 4 gr. of cream were uniformly distributed on a 10 × 10 cm sterile gauze, and then applied onto the lesion as for Fitostimoline gauzes. All the study drugs were applied once daily, starting from the baseline visit. When the burn dressing was replaced, any necrotic or exudate material was removed using a clip or buffer with saline solution. The surgical debridement could be performed only by the Investigator at the clinic. Follow-up visits were scheduled at 5-day intervals of therapy, for a maximal observational period of 20 days and a maximum of five visits. 

 In the case of lesion healing prior to the final visit and whenever a premature treatment discontinuation occurred, an early withdrawal visit was scheduled, if necessary. Treatment with antineoplastics, immunosuppressants, corticosteroids (local or systemic), or local cicatrising agents was not permitted. Antibiotics were permitted when necessary for the characteristics of the burn. The use of paracetamol (500 mg tablets) was also permitted for pain relief.

### 2.3. Outcome Measures

The percent reduction from baseline in the largest cross-diameter of the burn after 10 days of therapy was the primary efficacy variable. The reduction of the largest cross-diameter at the other time points, and of the surface area of the lesion, was also measured. After wound detersion, a film of transparent and sterile synthetic material was applied on the lesion, on which a second sheet of transparent synthetic material was spread down; the border of the lesion was traced on this sheet using an indelible pen (printing). The reference points used for the measurement of transversal diameters were then marked on the lesion print border. A photocopy of the printing was used for the computerized and centralised calculation of the lesion perimeter and surface area: the Visitrak portable digital system (Smith&Nephew, Milan, Italy) was used to track the lesion sizes. 

A related endpoint was disappearance (healing) of the lesion, corresponding to 0 values of cross-diameter and surface area, which was used to calculate the proportion of patients healed and time to healing. 

 Signs (functional limitation due to pain; perilesional erythema, oedema) and symptoms (burning, pain at rest, pain at medication, itching) due to the lesion were measured using a 5-point scale (0 to 4: absent, mild, moderate, severe, very severe) to obtain a total symptoms score (TSS) ranging from 0 (absence of symptoms) to 28 (maximal symptoms). Other efficacy variables included: clinical success at the last visit (defined as healing of the lesion or a reduction of at least 50% of baseline TSS); the percent of reepithelialisation (reported as ≤25%; >25% and ≤50%; >50% and ≤75%; >75% and ≤95%; >95% of initial lesion); and the use of relief paracetamol. The patient's acceptance of study drug in terms of ease of use and convenience was expressed using a 4-point scale (0 to 3: poor, sufficient, good, very good). Adverse events were recorded at any time throughout the study.

### 2.4. Ethics

Informed consent was signed by all participants prior to the start of any study-related procedure. The study protocol was approved by the reference Ethic Committee of each participating site.

### 2.5. Statistical Analysis

The sample size was based on a hypothesis of equivalence between the three treatments in the primary efficacy variable. The percent reduction from baseline to Day 10 in the largest burn diameter was expected to be in a range of 65–85%, equivalent to 0.938 to 1.173 in the angular scale where the distribution was likely to be approximately normal, and the standard deviation was assumed to be around 0.30 in this scale. Once defined alpha = 0.05 two-sided and power = 0.80, 120 evaluable patients in each arm were calculated to be required to rule out a clinically relevant difference of 10% or more (in the original untransformed scale) in the primary endpoint between Fitostimoline soaked gauzes and cream. The same number of patients had to be enrolled in the catalase gel arm, in order that the test power for comparison with the combined Fitostimoline arms was 0.90.

The following populations were considered for data analysis: safety, that is, all randomised patients who received at least one application of study medication; intention-to-treat (ITT), that is, all patients of the safety population who did not violate major inclusion-exclusion criteria; efficacy, that is, all patients of the ITT population who were visited at least once after the baseline visit; per protocol (PP), that is, all patients of the efficacy population who completed the study without major violations of study procedures.

The primary analysis of efficacy was performed on the reduction (as percent of baseline) in the largest burn diameter at Day 10 after angular (arcsine of square root) transformation of data. Nonparametric methods (Kruskal-Wallis and Mann-Whitney tests, Hodges-Lehmann estimator of location shift with Moses CIs) were used in the comparisons between groups as the variable distribution departed from normal (gaussian) even after transformation. The Mann-Whitney test for independent groups was used to compare Fitostimoline soaked gauzes with Fitostimoline cream. Since the two formulations resulted as clinically equivalent (i.e., the 95% CI of the Hodges-Lehmann median difference was entirely contained within an interval of ±10%), the data from both Fitostimoline groups were pooled and compared with catalase gel using Student's *t*-the Mann-Whitney test. 

The same methods described for the primary efficacy analysis were used for the reduction (as percent of baseline) in the largest burn diameter at the other time points Day 5 and in the burn surface area at Day 5 and at Day 10. At Day 15 or at end of study the vast majority of the burns were healed; therefore, the lesion status was analysed as a binary variable (present/absent) by means of relative risk estimates and Fisher's exact test.

Percent of reepithelialisation, paracetamol consumption, TSS (both as absolute values and as percent reduction from baseline) and the scores of the individual signs and symptoms were compared between groups using nonparametric tests (Kruskal-Wallis and Mann-Whitney). 

The last observation was carried forward in the primary efficacy analysis and in all secondary analyses at fixed timepoints, that is, at Day 10 or at end of study.

Survival analysis methods (i.e., Kaplan-Meier estimates and log rank test) were used to compare time to healing and time to reepithelialisation >95%. Data concerning patient's opinion of acceptance were analysed using nonparametric tests (Mann-Whitney and Kruskal-Wallis).

## 3. Results

### 3.1. Patient Disposition and Baseline Characteristics

A total of 227 patients (mean age 41.3 years, 52% females) were enrolled at eight Italian hospital Units and were randomised to receive the assigned treatment: 77 (33.9%) were included in the Fitostimoline soaked gauzes group, 73 (32.2%) in the Fitostimoline cream group and 77 (33.9%) in the catalase gel group. All randomised patients entered the safety and ITT population. A total of 209 patients, 74 (96.1% of randomised) in the Fitostimoline gauzes group, 69 (94.5%) in the Fitostimoline cream group and 66 (85.7%) in the catalase gel group, were treated up to Day 20 or healing, while 8 patients among those visited at least once after randomisation discontinued the treatment due to consent withdrawal or unattended follow-up visits (1 in the Fitostimoline gauzes group, 2 in the Fitostimoline cream group and 5 in the catalase gel group). Nineteen patients included in the completer population, 1 in the Fitostimoline gauzes group, 7 in the Fitostimoline cream group and 11 in the catalase gel group, had major protocol violations (mainly due to poor compliance to treatment schedule) and were excluded from the PP population. 

Table 1 shows the demographic and main baseline characteristics in the three groups. There were no substantial differences between groups for any of the measured demographic parameters, for sizes of the lesions, and for TSS. More than half of the burns (55.1% in the total population) were caused by hot aqueous liquid or steam, and most were located at the limbs, especially at the upper extremities (58.1% overall).

### 3.2. Efficacy

**Figure 1:**
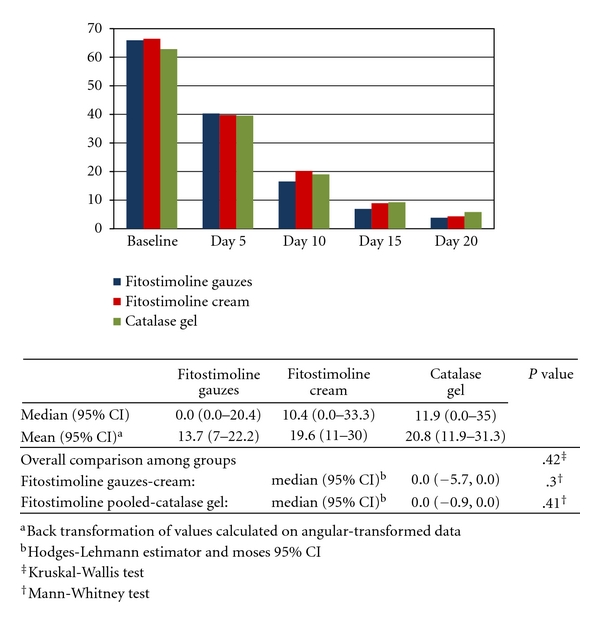
Mean values of largest burn diameter (mm) during the study and results of comparisons between groups at Day 10 (efficacy population).

Figure 1 shows the largest burn diameter at any time point in the efficacy population (i.e., patients of the ITT population who were visited at least once after the baseline) and the comparisons between groups at Day 10 (primary endpoint). The mean largest burn diameter progressively decreased from baseline to end of study in all treatment groups. There were no statistically significant differences in the overall comparison between groups between the two Fitostimoline forms, and between the pooled Fitostimoline groups and catalase gel, in values expressed as percent ratio of the baseline value. The difference between the Fitostimoline soaked gauzes group and the Fitostimoline cream group did not exceed 10% of baseline in either direction, which proved that the two forms were essentially equivalent, as well as this result was obtained in the comparison between the pooled Fitostimoline groups and catalase gel. No statistically significant differences between groups were also observed at Day 5.

**Figure 2:**
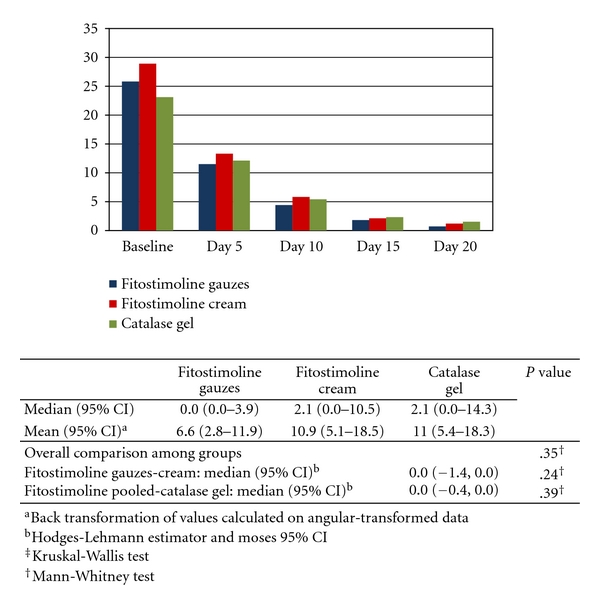
Mean values of lesion surface area (cm^2^) during the study and results of comparisons between groups at Day 10 (efficacy population).

The lesion surface area at any time point in the efficacy population and the comparisons between groups at Day 10 are shown in Figure 2. Mean lesion surface area progressively decreased from baseline in all treatment groups. The comparisons between groups did not show statistically significant differences at the time points examined between the two Fitostimoline forms, and between the pooled Fitostimoline groups and catalase gel, in values expressed as percent ratio of the baseline value. The results of largest burn diameter and lesion surface area in the PP population did not differ from those observed in the efficacy population.

The analysis of lesion size (both the largest cross-diameter and the surface area) at end of study in the efficacy population (Table 2) did not show significant differences between the two Fitostimoline groups in terms of lesion disappearance (size 0 equivalent to complete healing), whereas the risk of not achieving lesion disappearance was significantly lower in the pooled Fitostimoline groups compared to the catalase gel group (*P* = .020, Fisher's exact test).

The median time to healing was 11 days in the Fitostimoline gauzes group, 12 days in the Fitostimoline cream group and 12 days in the catalase gel group, without significant differences between groups.

Clinical success (i.e., reepithelialisation >95% or TSS reduction ≥50% from baseline) at the last visit in the efficacy population was obtained by 74 (98.7%) in the Fitostimoline gauzes group, 69 (97.1%) in the Fitostimoline cream group and 68 patients (95.8%) in the catalase gel group. In the PP population, only one patient in the Fitostimoline cream group (1.6%) and none in the other two arms were failures according to this definition.

The results of reepithelialisation at Day 10 in the efficacy population are presented in Table 3. The proportion of patients who achieved the various degrees of reepithelialisation was comparable in the three treatment groups: a rate >75% of reepithelialisation was observed in 51 patients (68.0%) in the Fitostimoline gauzes group, in 46 (64.8%) in the Fitostimoline cream group and in 47 (66.2%) in the catalase gel group. Although the proportion of patients with >95% of reepithelialisation was higher in the Fitostimoline cream group than in the other two groups, the comparisons between groups did not show statistically significant differences, as well as there were no differences between groups at end of study. The median time to reepithelialisation >95% was 11 days in the Fitostimoline gauzes group, 12 days in the Fitostimoline cream group and 11 days in the catalase gel group.

**Figure 3:**
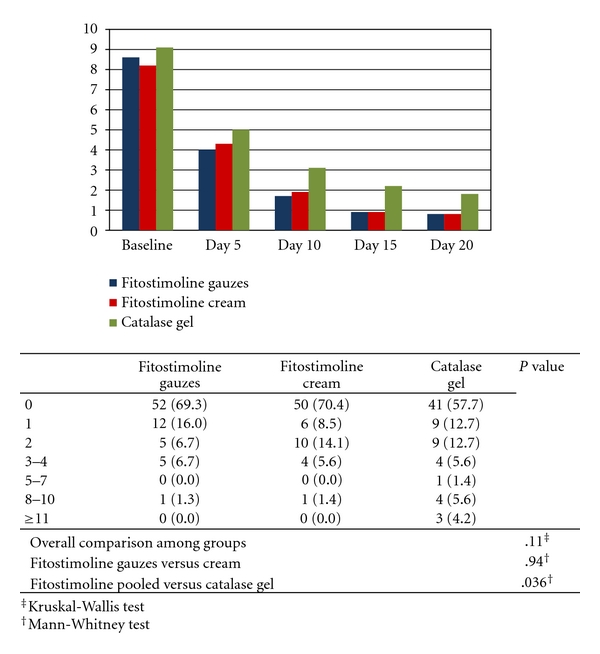
Mean values of total symptoms score during the study and results of comparisons between groups at end of study (efficacy population).

Figure 3 shows the results of TSS at any time point in the efficacy population and the comparisons between groups at end of study. A progressive decrease in mean TSS from baseline to end of study was observed in all groups. The comparisons between groups at end of study did not show significant differences between the two Fitostimoline groups, while a statistically significant difference was found in favour of the pooled Fitostimoline groups compared to the catalase gel group (*P* = .036, Mann-Whitney test).

In the analysis of TSS change from baseline expressed in terms of increase, no change and different degrees of decrease (<50%, 50–99% and 100% regression), there were no statistically significant differences between groups at both Day 10 and end of study.

The median time to TSS decrease ≥50% from baseline was 6 days in the Fitostimoline gauzes group, 7 days in the Fitostimoline cream group and 7 days in the catalase gel group. 

 In the analysis of individual signs and symptoms (PP population), a statistically significant difference between the pooled Fitostimoline groups and catalase gel was observed for pain at medication (*P* = .021, Mann-Whitney test), pain at rest (*P* = .007) and burning (*P* = .027) at end of study due to a higher proportion of patients free of the symptom in the pooled Fitostimoline groups (94.1% versus 83.6% for pain at medication, 100% versus 94.5% for pain at rest, 97.0% versus 89.1% for burning). There were no significant differences in the analysis of the other signs and symptoms, as well as in all parameters between the two Fitostimoline forms.

The rate of patients who took paracetamol during the study was comparable in the three treatment groups: 16.0% in the Fitostimoline gauzes group, 11.3% in the Fitostimoline cream group and 15.5% in the catalase gel group. None of patients in any group required surgical cleaning or debridement of the lesion at any time during the study.

### 3.3. Acceptability

Among the patients in the efficacy population who expressed an opinion on treatment acceptability, the proportion with very good or good opinion was higher in the Fitostimoline soaked gauzes group (69 of 71, 97.2%) and in the Fitostimoline cream group (63 of 66, 95.5%) than in the catalase gel group (59 of 66, 89.4%), while a poor opinion was reported by 4 patients (6.1%) in the catalase group and by none in the two Fitostimoline groups. The overall difference between the pooled Fitostimoline groups and the catalase gel group was statistically significant (*P* = .006, Mann-Whitney test).

### 3.4. Safety

The rate of patients with adverse events was higher in the catalase gel group (4 patients, 5.2%) than in patients treated with Fitostimoline (only 1 patient—1.3%—in the Fitostimoline gauzes group). The adverse event in the patient in the Fitostimoline gauzes group consisted of local signs of infection. In the catalase gel group, 3 adverse events consisted of application site reactions (itching in 1 case, burning upon application in 1 and itching and erythema in 1) and 1 adverse event consisted of abdominal pain.

## 4. Discussion

The dermatological application of the aqueous extract of *Triticum vulgare*, that is, the active ingredient of Fitostimoline-based products, is largely used in Italy from approximately 20 years in the reparation of cutaneous lesions and in all conditions requiring epithelial restoring. The availability on the market of different formulations of Fitostimoline allows their use in different affections and variables sizes of lesions, as well as according to the patients' acceptance and preference. In a double-blind, randomised, controlled study [11], the efficacy of Fitostimoline gauzes in the treatment of second-degree burns resulted to be significantly superior to that of placebo gauzes. Another recent placebo-controlled study [12] that included 200 patients with evidence of ulcerated dystrophic skin lesions and delayed cicatritial healing showed a significantly increased rate of tissue repair and a rapid remission of clinical symptoms in the Fitostimoline-treated group compared to placebo. Fitostimoline in form of cream also proved to be effective in the treatment of gynaecological affections [13]. In this study we have compared the effects of two different formulations of Fitostimoline (soaked gauzes and cream) with a gel form of equine catalase (Citrizan) in the topical treatment of second-degree burns of small to medium size. Citrizan gel contains equine catalase and is indicated in the topical treatment of burns, sores and ulcers. The catalase is a haemoproteic enzyme that exerts a peroxidase activity and also allows the release of molecules of oxygen in the injured tissue. Burns and trauma are associated with increased free radical production, which contribute to the imbalance in endogenous antioxidant capacity and the extension of primary lesions [14]. The role of antioxidants as micronutrient in the regeneration and recovery of burn lesions is well established [15]. It has been reported that cellular oxidative stress is a critical step in burn-mediated injury; therefore, antioxidant strategies designed to either inhibit free radical formation in the necrobiotic tissue or to scavenge free radicals may provide organ protection in patients with burn injury [16]. For these reasons, we have considered that equine catalase is a reliable and validated active comparator in testing of pharmacological agents in the reparation of second-degree burns.

The efficacy results of this study showed that treatment with both Fitostimoline formulations and catalase gel was associated with a marked improvement from baseline of the primary efficacy variable (largest cross-diameter of the burn at Day 10) in patients with second-degree superficial cutaneous burn of thermal origin, without statistically significant differences in the comparison between Fitostimoline gauzes and cream, and between pooled Fitostimoline groups and catalase gel.

Changes from baseline of largest cross-diameter of the burn at the other examined time points also did not differ between groups. All investigational study drugs also produced a progressive and similar decrease from baseline of the surface area of the lesion, without statistically significant differences between groups. However, at end of study the rates of healing in the groups receiving Fitostimoline were higher in the pooled Fitostimoline groups than in catalase gel group. Although rates of healing of burn lesions and of reepithelialisation >95% in the efficacy population were generally higher in the Fitostimoline soaked gauzes and in the Fitostimoline pooled groups than in the catalase gel group, the comparison between groups of time to healing and time to reepithelialisation >95% did not show statistically significant differences. With this respect, it is likely that the lower than scheduled sample of patients may have limited the possibility to detect significant differences between groups.

Improvements in total symptoms score at end of study were significantly more marked in the Fitostimoline groups than in the catalase gel group, as a result of a better outcome in pain at medication, pain at rest and burning. Only a minority of patients (which was similar in all groups) required rescue paracetamol and none required surgical or detersion procedures of the lesions. The safety results of this study showed that both formulations of Fitostimoline were at least as safe and well tolerated in terms of local and general adverse effects as the reference catalase gel formulation. As confirmation of the favourable efficacy and tolerability profile of Fitostimoline, a significantly better opinion on acceptability of treatment, which is mainly based on subjective symptoms and tolerability, was reported by patients treated with Fitostimoline formulations compared to those treated with catalase gel.

Although some differences in favour of Fitostimoline over catalase were observed after more than 10 days of treatment, caution should be used in the interpretation of these results. In this study, *P*-values for secondary analyses should not be interpreted at the conventional significance level of  .05 as they arise in a frame of repeated measures and multiple testing. Furthermore, some of the patients who did not heal or improve were not clinical failures at study conclusion but were rather lost to observation, the disease status at the time of discontinuation being carried forward according to the planned analysis. Thus, the worse results observed with catalase might be partly due to the higher rate of study discontinuations in this group.

It should also be considered that, although measurements of lesions were centralised in blinding condition, the study used an open-label design due to the topical administration of the three study medications, thus making the double dummy design (use of the placebo of all pharmaceutical forms) inapplicable, which cannot exclude some degree of bias in secondary subjective endpoints (e.g., symptoms).

In conclusion, the results of this study have shown that the topical application of Fitostimoline in soaked gauzes and cream form is at least as effective and safe as a gel form containing equine catalase in the treatment of second-degree superficial cutaneous burn of thermal origin.

## Figures and Tables

**Table 1:** Demographic and lesion baseline characteristics in the three groups (all patients enrolled). Entries are mean ± standard deviation, unless otherwise stated.

	Fitostimoline soaked gauzes	Fitostimoline cream	Catalase gel
Sex, *N* (%) of females	41 (53.2)	35 (47.9)	42 (54.5)
Age (years)	40.5 ± 15.0	42.8 ± 14.0	40.8 ± 15.3
Weight (Kg)	69.7 ± 13.2	73.0 ± 14.1	70.1 ± 13.5
Height (cm)	169 ± 9	170 ± 9	169 ± 9
Body mass index (Kg/cm^2^)	24.3 ± 4.0	25.2 ± 4.2	24.4 ± 3.9
Largest diameter (cm)	65.9 ± 20.8	66.4 ± 23.8	62.8 ± 20.0
Lesion surface area (cm^2^)	25.8 ± 16.2	28.9 ± 20.3	23.1 ± 15.0
Total symptoms score	8.6 ± 3.7	8.2 ± 3.5	9.1 ± 4.2

**Table 2:** Lesion size and lesion disappearance (healing) at end of study in the efficacy population.

	Fitostimoline soaked gauzes	Fitostimoline cream	Catalase gel	Fitostimoline pooled
Largest cross-diameter^#^				
mean (95% CI)^a^	0.1 (0.0–0.5)	0.4 (0.0–1.5)	2.3 (0.3–5.9)	0.2 (0.0–0.7)

Surface area^#^				
mean (95% CI)^a^	0.1 (0.0–0.3)	0.2 (0.0–0.7)	1.6 (0.1–4.5)	0.1 (0.1–0.3)

*N* (%) of values = 0	71 (97.3)	65 (91.5)	60 (84.5)	136 (94.5)
*N* (%) of values > 0	2 (2.7)	6 (8.5)	11 (15.5)	8 (5.5)

Overall comparison among groups				*P* = .024*
Fitostimoline gauzes/cream	RR (95% CI)^b^ 0.32 (0.066–1.51)			*P* = .16*
Fitostimoline pooled/Catalase gel	RR (95% CI)^b^ 0.35 (0.15–0.84)			*P* = .020*

^#^As percent ratio of baseline value.

^a^Back transformation of values calculated on angular-transformed data.

^b^Relative risk of not achieving lesion disappearance (size 0).

*Fisher's exact test.

**Table 3:** Reepithelialisation at Day 10 (efficacy population).

	Fitostimoline soaked gauzes	Fitostimoline cream	Catalase gel	Fitostimoline pooled
Range	*N* (%)	*N* (%)	*N* (%)	*N* (%)

>0% to 25%	3 (4.0)	4 (5.6)	5 (7.0)	7 (4.8)
>25% to 50%	11 (14.7)	6 (8.5)	13 (18.3)	17 (11.6)
>50% to 75%	10 (13.3)	15 (21.1)	6 (8.5)	25 (17.1)
>75% to 95%	9 (12.0)	11 (15.5)	13 (18.3)	20 (13.7)
>95%	42 (56.0)	35 (49.3)	34 (47.9)	77 (52.7)

Overall comparison among groups			*P* = .64^‡^	
Fitostimoline gauzes versus cream			*P* = .62^†^	
Fitostimoline pooled versus catalase gel			*P* = .42^†^	

^‡^Kruskal-Wallis test.

^†^Mann-Whitney test.
